# Specific and Sensitive Diagnosis of *BCOR*-ITD in Various Cancers by Digital PCR

**DOI:** 10.3389/fonc.2021.645512

**Published:** 2021-02-25

**Authors:** Doriane Barets, Romain Appay, Marie Heinisch, Maxime Battistella, Corinne Bouvier, Guillaume Chotard, François Le Loarer, Nicolas Macagno, Romain Perbet, Daniel Pissaloux, Audrey Rousseau, Arnaud Tauziède-Espariat, Pascale Varlet, Alexandre Vasiljevic, Carole Colin, Frédéric Fina, Dominique Figarella-Branger

**Affiliations:** ^1^ APHM, CHU Timone, Service d’Anatomie Pathologique et de Neuropathologie, Marseille, France; ^2^ Aix-Marseille Univ, CNRS, INP, Inst Neurophysiopathol, Marseille, France; ^3^ Department of Pathology, Hôpital Saint-Louis, Assistance Publique-Hôpitaux de Paris, Université de Paris, Inserm U976, Paris, France; ^4^ Service de Pathologie, Groupe Hospitalier Pellegrin, CHU de Bordeaux, Bordeaux, France; ^5^ Department of Pathology, Institut Bergonié, Bordeaux, France; ^6^ Institute of Pathology, CHU Lille, Lille, France; ^7^ LilNCog, Lille Neuroscience and Cognition, Univ. Lille, Inserm, CHU Lille, U1172, Lille, France; ^8^ Department of Translational Research and Innovation, Léon Bérard Cancer Center, Lyon, France; ^9^ Claude Bernard University Lyon 1, INSERM 1052, CNRS 5286, Cancer Research Center of Lyon, Centre Léon Bérard, Lyon, France; ^10^ Département de Pathologie Cellulaire et Tissulaire, CHU Angers, Angers, France; ^11^ Department of Neuropathology, GHU Paris-Psychiatrie Et Neurosciences, Sainte-Anne Hospital, Paris, France; ^12^ Centre de Pathologie Est, Groupement Hospitalier Est, Hospices Civils de Lyon, Bron, France; ^13^ ID Solutions, Research and Development, Grabels, France

**Keywords:** digital PCR assay, *BCOR*-internal tandem duplication, diagnostic marker, HGNET-BCOR, FFPE tissue

## Abstract

*BCOR* is an epigenetic regulator altered by various mechanisms including *BCOR*-internal tandem duplication (*BCOR*-ITD) in a wide range of cancers. Six different *BCOR*-ITD in the 3’-part of the coding sequence of exon 15 have been reported ranging from 89 to 114 bp in length. *BCOR*-ITD is a common genetic alteration found in clear cell sarcoma of the kidney and primitive myxoid mesenchymal tumor of infancy (PMMTI) and it characterizes a new type of central nervous system tumor: “CNS tumor with *BCOR*-ITD”. It can also be detected in undifferentiated round cell sarcoma (URCS) and in high-grade endometrial stromal sarcoma (HGESS). Therefore, it is of utmost importance to search for this genetic alteration in these cancers with the most frequent technique being RNA-sequencing. Here, we developed a new droplet PCR assay (dPCR) to detect the six sequences characterizing *BCOR*-ITD. To achieve this goal, we used a single colored probe to detect both the duplicated region and the normal sequence that acts as a reference. We first generated seven synthetic DNA sequences: ITD0 (the normal sequence) and ITD1 to ITD6 (the duplicated sequences described in the literature) and then we set up the optima dPCR conditions. We validated our assay on 19 samples from a representative panel of human tumors (9 HGNET-BCOR, 5 URCS, 3 HGESS, and 2 PMMTI) in which *BCOR*-ITD status was known using at least one other method including RNA sequencing, RT-PCR or DNA-methylation profiling for CNS tumors. Our results showed that our technique was 100% sensitive and specific. DPCR detected *BCOR*-ITD in 13/19 of the cases; in the remaining 6 cases additional RNA-sequencing revealed *BCOR* gene fusions. To conclude, in the era of histomolecular classification of human tumors, our modified dPCR assay is of particular interest to detect *BCOR*-ITD since it is a robust and less expensive test that can be applied to a broad spectrum of cancers that share this alteration.

## Introduction

BCL-6 transcriptional corepressor (*BCOR*) gene is located at Xp11.4 and comprises 16 exons encoding a ubiquitously expressed transcriptional repressor (1, 2). The principal isoform, encoded by 14 exons, gives rise to a protein of 1,755 amino acids. BCOR protein contains two main functional binding domains. The BCL-6 binding domain allows binding to the POZ domain of BCL-6 and increases its function as a repressor of transcription (2). The other domain is the polycomb-group RING finger homolog (PCGF) ubiquitin-like fold discriminator (PUFD), a domain binding to some of the PCGF proteins forming repressive complexes involved in epigenetic histone modification. BCOR is part of one of the six currently described non-canonical variants of the polycomb repressive complex 1, the PRC1.1 (3).

Epigenetic regulators are potential proto-oncogenes or tumor suppressors, depending on the function of their target genes. *BCOR* alterations are reported in different human cancers, with a key role in neoplastic transformation or in tumor progression. Two major genetic alterations have been reported: gene fusions (mainly *BCOR-CCNB3*, *BCOR-MAML3*, and *ZC3H7B-BCOR*) and internal tandem duplications (ITD) of the PUFD domain (*BCOR*-ITD). *BCOR* gene fusions have been mainly recorded in undifferentiated round cell sarcoma (URCS), high-grade endometrial stromal sarcoma (HGESS) and ossifying fibromyxoid tumors (4–8). *BCOR*-ITD is a common genetic alteration detected in clear cell sarcoma of the kidney (CCSK), primitive myxoid mesenchymal tumor of infancy (PMMTI) and in some central nervous system (CNS) high-grade neuroepithelial tumor (HGNET) first reported as CNS HGNET-BCOR (9–14). CNS HGNET-BCOR have been discovered after DNA-methylation profiling of a large series of CNS tumors first diagnosed as “primitive neuroectodermal tumors” (14). Most of these tumors displayed *BCOR* internal tandem duplication. The C-IMPACT-NOW group recommends retaining “CNS tumor with *BCOR* internal tandem duplication” as a new tumor type ((15); WHO classification of CNS tumors, in progress). It is of note that some tumors such as CCSK, soft tissue URCS, or HGESS may display either *BCOR* fusion or *BCOR*-ITD although the latter alteration is more common (3). Interestingly, strong immunohistochemical nuclear expression of BCOR protein is highly suggestive of *BCOR*-ITD (12, 16, 17). However in some cases the immunostaining might be faint or absent and genetic analysis is mandatory to confirm or rule-out *BCOR*-ITD. Lastly, different missense, non-sense, frameshift, and insertion/deletion mutations have been described in a large variety of human tumors (reviewed in (3)).

Integration of molecular alterations to define tumors brings new challenges for the pathologist to make the best use of a precious limited tissue specimen for molecular studies. Such investigations are inexorably costly, time and sample-consuming, and the sensitivity of various approaches might be insufficient in a context of a prominent background of non-tumoral DNA. Internal tandem duplications of *BCOR* exon 15 (*BCOR*-ITD) are highly suggestive of some histological subtypes especially in the pediatric population and is mandatory for the diagnosis of CNS tumors with *BCOR*-ITD. Among the different methods available to identify gene alterations, the digital PCR (dPCR) constitutes a very interesting strategy as it is a rapid, cost-effective, and very sensitive tool. Digital PCR is based on the limit dilution of a DNA sample by fractionating and randomly allocating the DNA into reaction chambers (5,000 to 8,000,000) *via* a water-in-oil emulsion process or any other reservoir used as a reaction chamber. A PCR reaction takes place in each of the reaction chambers in the presence of a probe or DNA intercalator whose fluorescence emission is specific to the presence of the genomic target. Following this amplification reaction, a fluorescence signal (color) is produced (1) or not (0) and analyzed for each chamber, allowing a binary result. By counting the positive and negative chambers, and applying Poisson’s law, it is possible to calculate the real number of targets per chamber and to quantify in an absolute way the concentration of the genomic target in the DNA sample.

We have successfully used this technique for the detection of recurrent genome copy number variations (CNV) including duplications in CNS tumors, using, in the same DNA test sample, a standard dPCR design with two different colored probes directed against a target sequence and a reference sequence (invariable on the same chromosome, same arm or another chromosome or SNP) (18–20). However, the duplicated region of the *BCOR*-ITD is very small, hindering its segregation into different droplets and its CNV evaluation by standard designs.

Here we report a new approach that uses a single colored probe to detect both the duplicated region and the normal sequence that acts as a reference. Specifically, we developed a dPCR method that detects the six sequences found in the *BCOR*-ITD described to date.

## Material and Methods

### Design of the BCOR-ITD dPCR Detection Assay

Six different BCOR-ITD alterations (ITD1, ITD2, ITD3, ITD4, ITD5, and ITD6) with tandem duplications of 89 to 114 bp in the 3′ part of the coding sequence of exon 15, with or without insertion of 1 or 2 bp between the duplicated sequences have been described (9). Duplications involve the 167 bases located between the coordinates chrX: 4073112-40052111 (hg38), and are composed of 30 to 38 amino acids. ITDs involve amino acids 1.701–1.755 (ENST00000378444.9) in the C-terminal PUFD domain of the protein (2). Fourteen successive amino acids have been systematically found to be duplicated in the six isoforms: LDLVEFTNEIQTLL (p.L1721_L1737), they are encoded by 42 bases: TTA GAT CTG GTG GAA TTC ACG AAC GAA ATT CAG ACT CTG CTG (c.5170-5211).

**Figure 1:**
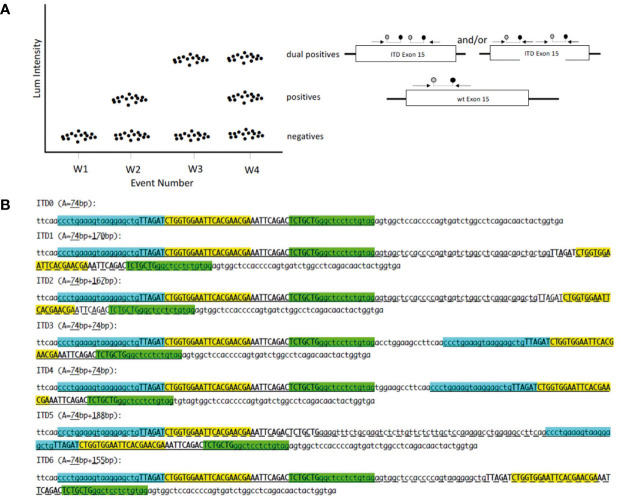
**(A)** dPCR for tandem duplication in one channel: W1 no template control; W2 normal sample; W3 homozygous ITD BCOR; W4 heterozygous ITD BCOR; forward primer; reverse primer; probe. **(B)** Position of primers and probe on synthetic sequences. ITD0: normal sequence; ITD1 to ITD6 sequences with internal tandem duplication (ITD). Forward primer in cyan; reverse primer in green; probe in yellow; invariable duplicated sequence in upper case; A: amplicon size; classic amplicon is underlined; long amplicon is underlined with dashes.

The dPCR assay that we has been developed is composed of a forward and a reverse primer as well as a chemically modified TaqMan^®^ type internal probe designed to amplify and target a sequence including the systematically duplicated region. The target sequence then becomes its own reference (**Figure 1A**). The expected amplicon measures 74 bp and contains the 42 pairs of duplicated bases common to the 6 ITDs. Since the internal fluorescent probe is complementary to 20 of the 42 bp common to the 6 ITDs, it might hybridize twice to the duplicate allele (**Figure 1B**). We postulate that based on the design of our detection assay, distinct PCR products should be obtained. For ITD3 and ITD4, the forward and reverse primers are perfectly complementary of two regions in the ITD: the normal sequence and its duplication and therefore two 74 bp amplicons might be generated. For the other four ITDs, only one of the primers hybridizes twice (the reverse primer in case of ITD1, ITD2, and ITD6 and the forward in ITD5) and therefore we expected to observe a larger amplicon in addition to the 74 bp amplicon (170, 167, 155, and 188 bp in ITD1, ITD2, ITD6, and ITD5 respectively) (**Figure 1B**).

### Generation of Synthetic DNA Sequences

In order to be sure that our assay will be able to detect the six different BCOR-ITD alterations described so far, we generated seven synthetic DNA sequences: ITD0 which corresponds to the normal sequence and ITD1 to ITD6 representing the duplicated sequences described in the literature (**Figure 1A**).

The artificial sequences were cloned and produced by GENEWIZ (Leipzig, Germany). They were inserted in the poly-linker of pUC57-Kan (2626 bp) with, for ITD0 an inserted fragment of 123 bp, ITD1 a fragment of 219 bp, ITD2 a fragment of 216 bp, ITD3 a fragment of 213 bp, ITD4 a fragment of 213 bp, ITD5 a fragment of 237 bp, and for ITD6 a fragment of 204 bp. The lyophilized plasmids were linearized using KpnI (Thermo Fisher Scientific, France). Stock solutions ITD0_p_, ITD1_p_, ITD2_p_, ITD3_p_, ITD4_p_, ITD5_p_, and ITD6_p_ (ITDs_p_) at 50 ng/µl were prepared in 200 µl of Poly A+ 10 ng/µl in TE buffer and then calibrated to 60 to 150 copies/µl dPCR of DNA.

### Set-Up of Optima dPCR Conditions

We verified the efficiency and specificity of the assay to detect the seven pure sequences (ITD0_p_ to ITD6_p_) as well as their dilution with the normal sequence. For the latest purpose, we created six pools (ITD1_50_ to ITD6_50_) by combining 50% of ITD0 stock solution and 50% of ITD1 to ITD6 stock solutions with TE-poly A+ buffer as solvent. The mixing ranges for analytical performance analysis for each isoform, ITD1_R_ to ITD6_R_, were prepared using the same solutions.

We tested different conditions of temperature, time, primer/probe concentration, and temperature ramps to define a single dPCR condition. The conditions were optimized to promote double hydrolysis from the longest amplicons. We used a CNS tumor with the *BCOR*-ITD as positive control.

Optimal conditions were obtained with a final reaction volume of 21 µl, containing 10.5 µl of mix (ddPCR Supermix, Bio-Rad, France), 0.375 µmol of forward primer (5’-CCCTGAAAGTAAGGAGCTGTTAGAT-3’), 1.5 µmol of reverse primer (5’-CTACAGAGGAGCCCAGCAGA-3’), and 0.250 µmol of probe (FAM-CTGGTGGAATTCACGAACGA-BHQ) for 8µl of DNA sample. The PCR temperature/time conditions are: 10’ to 95°C (1°C/s) initial denaturation, 40 PCR cycles comprising a 30’’ denaturation step at 95°C (1°C/s), and a hybridization/elongation step from 1’ to 65°C (1°C/s), with a final post-cycle step from 10’ to 98°C (1°C/s). The nanodroplets were produced manually using the QX200™Droplet Generator (Bio-Rad) following the recommendations of the supplier, the PCR reactions were performed on C1000 Touch Thermal Cycler (Bio-Rad), the droplet count using the QX200™ Droplet Reader, the analysis using the QuantaSoft™ Analysis Pro Software (Bio-Rad). Normal cell lines without *BCOR*-ITD (SW48 and H1650 cell lines), duplicated case (C+), and PCR controls were routinely included in each assay.

### Human Samples

We included 19 samples from a representative panel of human tumors with previously known BCOR genetic alterations. In all cases the BCOR genetic status was assessed by at least one genetic analysis (RNA sequencing, RT-PCR, or DNA-methylation profiling for CNS tumors). According to pathological diagnosis, our series comprised nine CNS embryonal tumors with pathological features suggestive of HGNET-BCOR (4/9 have been previously published (16, 21)), five URCS, three HGESS, and two PMMTI (**Table 1**).

**Table 1:** Clinical and biological characteristics of the 19 patients.

Patient ID	Num	Age at diagnosis	Tumor location	Diagnosis	BCOR status by IHC	*BCOR*-ITD status by dPCR	Fractional abundance value of duplication	Other analysis techniques	Methylation class and score	RNAseq result
BCOR_1	HHH1937159	17 months	Posterior fossa	HGNET-BCOR	Positive	**Duplicated**	33%	Methylome	CNS HGNET-BCOR (0.99)	
BCOR_2	HHH1607884	3 years	Posterior fossa	HGNET-BCOR	Positive	**Duplicated**	60%	Methylome* PCR	CNS HGNET-BCOR (0.861)	
BCOR_3	HHH1912032	13 years	Temporal lobe	HGNET-BCOR	Negative	Not Duplicated		Methylome RNAseq	CNS HGNET-BCOR (0.57)	Fusion *BCOR-EP300*
BCOR_4	HHH1821010	21 months	Posterior fossa	HGNET-BCOR	Positive	**Duplicated**	45%	Methylome	CNS HGNET-BCOR (0.99)	
BCOR_5	HHH1837233	8 years	Temporoparietal lobe	HGNET-BCOR	Positive (faint)	**Duplicated**	8%	RNAseq	/	
BCOR_6	HHH2009974	12 months	Frontoparietal lobe	HGNET-BCOR	Negative	**Duplicated**	30%	Methylome	CNS HGNET-BCOR (0.99)	
BCOR_7	HHH1332915	4 years	Posterior fossa	HGNET-BCOR	Positive	**Duplicated**	23%	Methylome* PCR	CNS HGNET-BCOR (0.738)	
BCOR_8	HHH1602661	7 years	Posterior fossa	HGNET-BCOR	Positive	**Duplicated**	24%	Methylome* PCR	CNS HGNET-BCOR (0.689)	
BCOR_9	HHH2013375	7 years	Supratentorial	HGNET-BCOR	Positive	Not Duplicated		Methylome RNAseq	CNS HGNET-BCOR (0.85)	Fusion *KDM2B-NUTM2*
BCOR_10	HHH1837234	57 years	Peritoneal	URCS	Positive (faint)	Not Duplicated		RNAseq	/	Fusion *BCOR-ZC3H7D*
BCOR_11	HHH1837235	14 years	Paravertebral	URCS	Positive (faint)	Not Duplicated		RNAseq	/	Fusion *BCOR-CCNB3*
BCOR_12	HHH1837236	15 years	Anckle	URCS	Negative	Not Duplicated		RNAseq	/	Fusion *BCOR-CCNB3*
BCOR_13	HHH1927750	26 years	Cerebellopontine angle	URCS	Positive	**Duplicated**	34%	RNAseq	/	*BCOR*-ITD
BCOR_14	HHH1802983	10 years	Parotide	URCS	Positive	Not Duplicated		RNAseq	/	Fusion *BCOR-CCNB3*
BCOR_15	HHH1837237	58 years	Uterus	HGESS	Negative	**Duplicated**	14%	RNAseq	/	*BCOR*-ITD
BCOR_16	HHH1835204	43 years	Retroperitoneal	HGESS	Positive (faint)	**Duplicated**	7%	RT-PCR	/	*BCOR*-ITD
BCOR_17	HHH1837239	26 years	Vertebral	HGESS	Negative	**Duplicated**	9%	RNAseq	/	*BCOR*-ITD
BCOR_18	HHH1618658	8 months	Thigh	PMMTI	Positive	**Duplicated**	19%	RNAseq	/	*BCOR*-ITD
BCOR_19	HHH1800109	8 months	Mesentery	PMMTI	Positive	**Duplicated**	20%	RNAseq	/	*BCOR*-ITD

*450k Classifier v11.0 Ref (16).

ITD, internal tandem duplication; dPCR, digital PCR; IHC, immunohistochemistry; RNAseq, RNA-sequencing; HGNET-BCOR, high-grade neuroepithelial tumor-BCOR; URCS,undifferentiated round cell sarcoma; HGESS, high-grade endometrial stromal sarcoma; PMMTI, primitive myxoid mesenchymal tumor of infancy.

For each case, either one formalin-fixed paraffin embedded (FFPE) block or unstained sections were available. Search for BCOR protein expression by immunohistochemistry was performed as previously described (16) when the FFPE block was available. Cases from Marseille were retrieved from the APHM tumor bank (22). For the other cases, BCOR immunostaining status was given by the pathologist who sent the case.

### DNA Extraction

After macro-dissection of the areas containing tumor cells, DNA was extracted from the FFPE tissues using the IDXTRACT-mag-FFPE kit (ID-Solutions, Grabels France) coupled to the IDEAL-32 automaton (ID-Solutions) following the recommendations of the supplier. DNA was qualified and quantified using DNA calibrated by an external standard assay using the IDQUANTq Kit (ID-Solutions) and the Mic^®^ quantitative PCR instrument (Bio Molecular Systems, Queensland, Australia). If necessary, the DNA was diluted or concentrated (Vivacon 500, Sartorius) in order to be able to perform a dPCR assay of 8 µl with 5 ng targeted quantity (70 copies/µl dPCR) of DNA. The dPCR reactions were performed according to the optimal reaction conditions. One case with *BCOR*-ITD was taken as positive control whereas one case without any BCOR genetic alteration was used as negative control and use for the set-up of the limit of detection of the assay.

## Results

### Validation of the Optima dPCR Conditions

**Figure 2:**
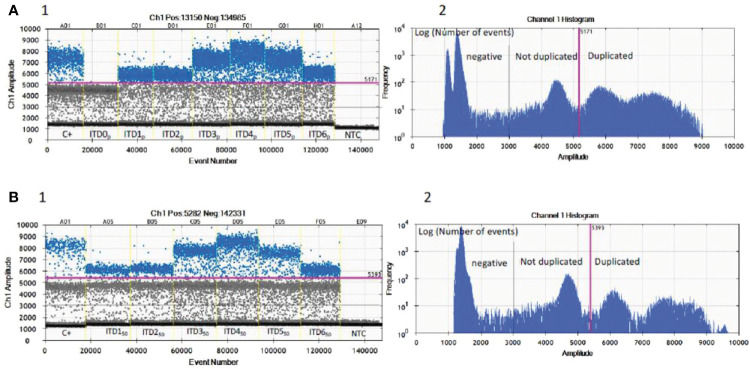
Validation of the optima dPCR conditions. Three droplet clusters differ according to their fluorescence levels: lowest signal (1,000< >3,000 AU) for negative droplets (below black line), “normal” cluster (3,000< >5,000 AU) have the same intensity level as ITD0_p_ (between black and pink line) and a third cluster of higher intensity (>5,000 AU) corresponding to a characteristic “*BCOR*-ITD” signal (above pink line). **(A**1**)** Nanodroplet cluster intensity for each pure artificial ITDs_p_ **. (A**2**)** Log of the number of events as a function of the amplitude of the fluorescence allows a single threshold to be set for all samples and isoforms (pink line). **(B**1**)** Nanodroplet cluster intensity for each six mixed ITDs_50_ with 50% of ITD0_p_. **(B**2**)** Log of the number of events as a function of the amplitude of the fluorescence allows a single threshold to be set for all samples and 50% mixed isoforms (pink line). C+, FFPE positive control; NTC, no template control; ITDs_p_, pure synthetic DNA sequences; ITDs_50_, 50% mixed ITD1_p_ to ITD6_p_ with ITD0_p_.

Under the defined dPCR conditions, we analyzed the signal intensities of the dPCR products using QuantaSoft software (Bio-Rad). **Figure 2A**1 shows that the signal height of the positive droplet cluster is strictly different from the negative droplet cluster as seen for the negative PCR control (NTC). There are differences in the intensities of the hydrolysis signal as a function of the artificial sequences (ITD0_p_ to ITD6_p_), ITD0_p_ having the lower intensity signal but strictly higher than the negative droplet cluster (>3,000 AU). The positive control (C+) has three droplet clusters that differ according to their fluorescence levels, with the lowest signal (1,000< >3,000 AU) for negative droplets, then a “normal sequence” cluster (3,000< >5,000 AU) of the same intensity level as ITD0_p_ and finally a third cluster of higher intensity (>5,000 AU) corresponding to a characteristic “*BCOR*-ITD” signal. From **Figure 2A**2, which represents the value of the log of the number of events as a function of the amplitude of the fluorescence, it is simple to set a threshold value of fluorescence allowing to distinguish ITD0_p_ from other ITD [ITD1_p to_ ITD6_p_ (>5000 AU)]. Above a value of 5,000 AU, the *BCOR* ITD1_p_ to ITD6_p_ are strictly different from ITD0_p_. Concerning the different *BCOR*-ITD sequences, ITD3_p_, ITD4_p_, and ITD5_p_ showed a higher intensity compared to the sequences ITD1_p_, ITD2_p_, and ITD6_p_. The analysis of the six mixtures ITD1_50_ to ITD6_50_ (**Figure 2B**1, 2) showed that the threshold value >5,000 AU makes it possible to distinguish in the same sample, the six artificial duplicated sequences from the normal sequence ITD0_p_. **Table 2** summarizes the different steps of the protocol, their duration as well as the potential pitfalls for each step.

**Table 2:** Summary of dPCR for *BCOR*-ITD detection with the different steps, their duration as well as the potential pitfalls for each step.

Steps	Duration	Potential pitfalls	Controls
Dewaxing and proteinase K digestion of FFPE tissues	4h or overnight digestion	Cross-contamination	0DEW
DNA extraction (IDXTRACT)	40min	Cross-contamination	0EXT
Qualification and quantification by QPCR (IDQUANTq)	1h30	Contamination, concentration<0.5ng/L (a), inhibition(b)	0QPCR, TPC Abnormal QPCR kinetic
Optional concentration (a)	1h15		
Optional dilution (b)	negligible		
*BCOR* dPCR assay	2h30	Contamination, contamination in ITD cluster> LoD(10), C+ with non-specific ITD signal, C_wt_ with specific ITD signal	C+, C_wt_, 0dPCR
Interpretation of dPCR	15min	Concentration<10 copies/µl (200copies or 0.5ng in assay), total droplet <5,000	

After macro-dissection of the areas of FFPE tissues containing tumor cells, dewaxing and proteinase K digestion were performed. DNA was extracted, quantified by QPCR, and then qualified before dPCR. If necessary the DNA should be concentrated to 0.5 ng/L DNA or diluted when QPCR kinetics was abnormal. Each potential pitfall has been secured by specific controls: 0DEW, 0EXT, 0QPCR and 0PCR which were all sample-free. 0DEW for dewaxing cross-contamination, 0EXT for DNA extraction, 0QPCR and calibrate true positive control (TPC) for QPCR, 0dPCR, ITD positive control (C+), and non-ITD Control (C_wt_) for dPCR. Technical validation was effective if: concentration>10 copies/µl dPCR (0.5ng in the assay), total droplet >5,000, C+ with specific ITD signal, C_wt_ without ITD signal and 0DEP, 0EXT, 0QPCR, and 0dPCR with contamination in ITD cluster< LoD(10).

### Analytical Performance

#### Limits of Detection

Determination of the limit of blank (LoB) and limit of detection (LoD; 95% CI) was evaluated by testing a non-ITD FFPE DNA sample containing 12.6 ng of DNA, 32 times in independent wells. In the absence of contamination, the positive cluster count in the duplicate-compatible fluorescence zone provides information on false positive events generated by the non-specific hydrolysis of the probe (LoB). The LoB obtained was four droplets. LoD was estimated using the statistical tool Gene-π from the www.gene-pi.com website. The LoD was 10 positive droplets (LoD =10) from 512,000 total droplets analyzed. Thus in theory, for 12.6 ng of total DNA analyzed, a sample is considered “*BCOR*-ITD” when there are more than 10 droplets forming a cluster above 5,000 AU.

#### Maximum Sensitivity

**Figure 3:**
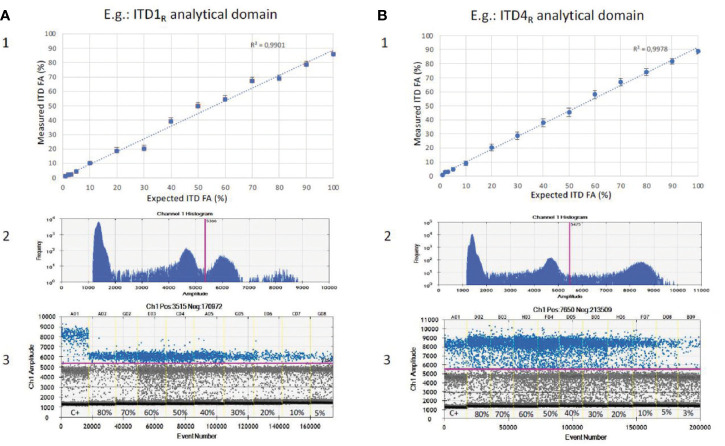
Sensitivity of *BCOR*-ITD detection. Results for serial dilutions of ITD1_R_ (**A**1, 2, 3) and ITD4_R_ (**B**1, 2, 3). **(A**1, **B**1**)** Linear correlation between expected fractional abundance of ITDs (x) and measured values (y). **(A**2, **B**2**)** The 1D plot showing the number of events for each fluorescence amplitude allowing to set a single threshold for all samples and isoforms (pink line). **(A**3, **B**3**)** The 1D plot showing for each dilution the positive events (*BCOR*-ITD) corresponding to the data points above the pink baseline marker, negative events at the bottom of the plot and reference events (*BCOR*-wildtype) between the two. C+, FFPE positive control; FA, fractional abundance.

In order to empirically evaluate the maximum sensitivity reachable for the detection of the six *BCOR*-ITD, we created mixing ranges (ITD_R_) covering fractional abundance from 100 to 1% of ITDs, with duplication increments of 10% between 100 and 10%, and four additional ranges at 5, 3, 2, and 1%. All the mixtures contained 5 ng of DNA (70 copies/µl dPCR) corresponding to the amount of DNA usually analyzed in routine practice. **Figure 3** illustrates results for ITD1_R_ and ITD4_R_ corresponding to the *BCOR*-ITD with respectively the lower and the higher intensity of the duplicated droplet clusters. The coefficient of determination R² calculated between the theoretical duplication percentages and the measured values was above 0.98 for all the ITD_s_. The lower fractional abundance permitting the distinction between the *BCOR*-ITD and the normal sequences was equal to 5% for ITD1 and ITD2, 3% for ITD3, ITD4, and ITD6 and 2% for ITD5. Thus, the maximum sensitivity of the assay was defined at 5% to detect all the ITDs.

### Validation of the *BCOR*-ITD Detection Assay in Human Samples

**Figure 4:**
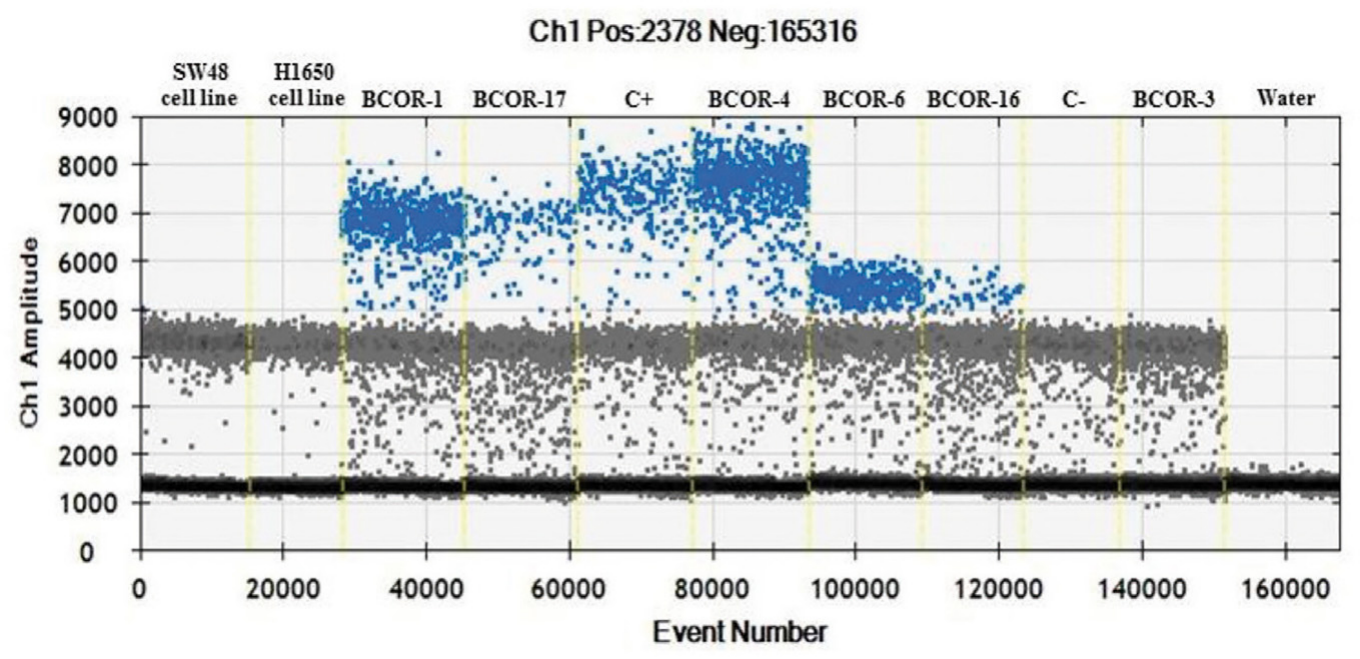
Example of *BCOR*-ITD detection by dPCR assay for six samples (BCOR-1, BCOR-17, BCOR-4, BCOR-6, BCOR-16, and BCOR-3). The 1D plot shows for each sample the positive events (*BCOR*-ITD) corresponding to the data points above the pink baseline marker, negative events at the bottom of the plot and reference events (*BCOR*-wildtype) between the two. Internal controls are analyzed at the same time: SW48 and H1650 cell lines which are *BCOR-wildtype*, duplicated and non-duplicated cases (C+ and C−), as well as NTC (no template control = water).

Among the nine cases that were classified as CNS embryonal tumors with pathological features suggestive of HGNET-BCOR, seven harbored duplication according to our *BCOR*-ITD dPCR detection assay. All these cases were classified by methylome analysis as CNS HGNET-BCOR (methylation score higher than 0.9 for the cases analyzed with the 850k, Heidelberg classifier v11b4 version, whereas the methylation score was lower in the three cases previously reported and confirmed by PCR (16); score ranging from 0.689 to 0.861, 450k methylation probe, classifier v11.0). Among these seven cases, 5/7 demonstrated BCOR nuclear immunoreactivity. Importantly, the two remaining cases that we also classified as CNS embryonal tumors with pathological features suggestive of HGNET-BCOR were negative for *BCOR*-ITD according to our *BCOR*-ITD dPCR detection assay. One case demonstrated BCOR nuclear immunopositivity in contrast to the other. These two cases also obtained a good score by DNA methylation analysis for CNS HGNET-BCOR although not high enough to retain the diagnosis of CNS HGNET-BCOR (score 0.57 and 0.85). These two cases demonstrated a fusion by RNA sequencing involving *BCOR* gene in one case (*EP300-BCOR* fusion) but not in the other (*KDM2B-NUTM2* fusion) (**Table 1**, **Figure 4**).

Among the five cases that were classified as URCS, only one was duplicated according to our *BCOR*-ITD dPCR detection assay. In this case, RNA sequencing also demonstrated *BCOR*-ITD and immunohistochemistry showed BCOR nuclei expression. In the four remaining cases that were not duplicated by dPCR, a fusion involving the BCOR gene was recorded by RNA sequencing. BCOR nuclei immunopositivity was recorded in 3 (although faint in two of them) whereas no staining was observed in the last case (**Table 1**).

At last, the five remaining cases (three HGESS and two PMMTI) that demonstrated *BCOR*-ITD by RNA sequencing or RT-PCR were found to be duplicated according to our *BCOR*-ITD dPCR detection system. BCOR nuclei immunopositivity was recorded in 3/5 cases (**Table 1**, **Figure 4**).

Besides, we observed in cases duplicated in our *BCOR*-ITD dPCR detection assay a fractional abundance ranging from 7 to 60%. The fractional abundance was higher in cases with pathological features suggestive of HGNET-BCOR that in other cases (Fisher exact test, p=0.029).

## Discussion

Recent advances in molecular characterization of human tumors have pointed out the role of the *BCOR* gene in tumorigenesis. *BCOR* is recognized as a gene fusion partner in a variety of mesenchymal neoplasms, whereas *BCOR–*ITD are found in up to 70% of CCSK and in some high grade endometrial sarcomas. It also characterizes two rare tumors occurring in infants or children: PMMTI and a rare CNS embryonal tumor first reported as HGNET-BCOR (14). Of importance and following cIMPACT–NOW 6 recommendations, the future WHO classification of CNS tumors will include «CNS tumor with *BCOR* internal tandem duplication» as a new tumor type (15). Therefore, it is of the utmost importance to use appropriate molecular tests to allow the detection of *BCOR*-ITD and thus make the correct diagnosis.

In this study, we describe the development and validation of a dPCR assay to detect this genetic alteration. The dPCR constitutes an interesting strategy to detect genetic alterations as it is a rapid, cost-effective and sensitive test with a short turnaround time. It is particularly useful on FFPE specimens since dPCR is a very robust approach on altered or fragmented DNA (23). In addition, dPCR limits the influence of enzyme inhibitors contained in formalin because their concentration is generally relatively low and their partitioning into only a few droplets has minimal influence on the analysis. Furthermore, it allows laboratories to obtain access to a unique, readily available tool to screen for various types of molecular alterations including mutations and CNV with an absolute quantification for each alteration (24). However dPCR has certain limitations: it only detects one molecular alteration (or few, in case of multiplex dPCR) in contrast to other techniques such as RNA-sequencing fusion panel, NGS fusion panel, or DNA-methylation profiling. Despite the increased cost, longer turnaround time, requirement for moderate, or even high amount of biological material, these techniques offer the benefit of delineating the complex genomic and epigenomic landscape of tumors, which may provide prognostic as well as therapeutic information. A more detailed discussion of the advantages and disadvantages of each technique have been highlighted previously (18).

Here we report a new approach that uses a single color to detect the duplication and the normal sequence that acts as a reference. This approach no longer assigns a binary enumeration of positive and negative signals in two colors, but detects and enumerates in the same color at greater intensity in chambers containing the duplicated sequence compared to the positive chambers without duplication. The target sequence then becomes its own reference. Using this technique, we have been able to develop a method for the detection by dPCR of the six sequences derived from the *BCOR*-ITD described to date. This disruptive approach to the use of the dPCR technique could be applied similarly for the detection of other ITD on genes of interest in the field of cancer (e.g. *FLT3*-ITD in *acute myeloid leukemia*).

When compared to RNA sequencing, RT-PCR or methylation profiling, used as “gold standard” techniques, this dPCR assay displays 100% specificity and sensitivity. On the other hand, nuclear accumulation of BCOR protein evaluated by immunohistochemistry was absent in 4/13 *BCOR*-ITD cases and present in 4 out of 6 cases that did not display *BCOR*-ITD. This is in contrast with previous studies including ours which reported strong BCOR nuclear accumulation in all HGNET-*BCOR*-ITD cases (16, 17). BCOR nuclear accumulation might also be absent in CCSK with *BCOR*-ITD since no immunostaining was recorded in 6 out of 54 CCSK *BCOR*-ITD cases in one study (25). BCOR nuclear accumulation is also a common feature in CCSK and HGESS harboring *YWHAE-NUTM2* fusion, a genetic alteration mutually exclusive from *BCOR*-ITD, suggesting that these two genetic alterations activate a common signaling pathway (5).

**Figure 5:**
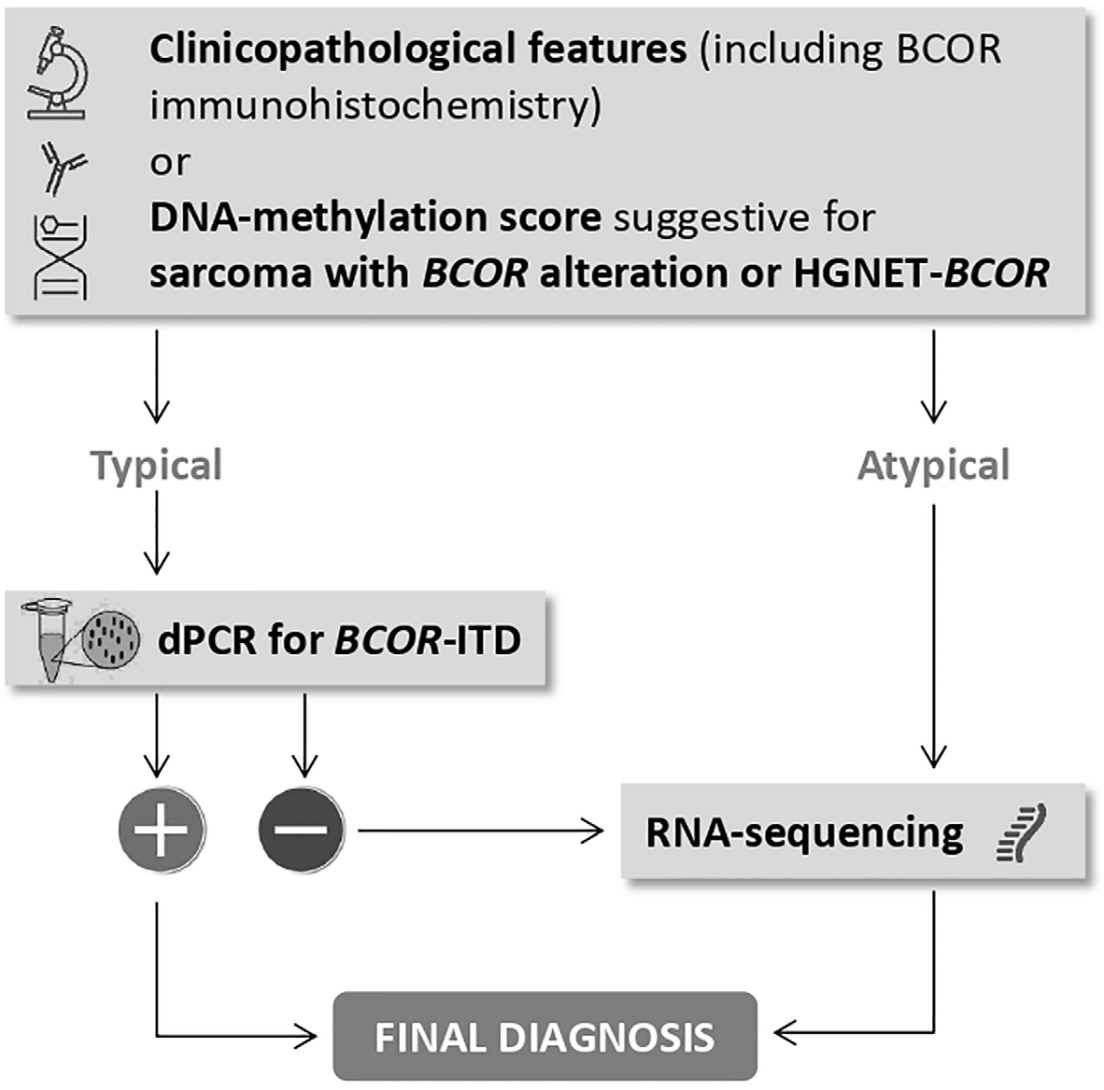
The place of dPCR for *BCOR*-ITD in the diagnostic workflow to reach a final diagnosis.

Of importance, diagnosis of CNS tumor with *BCOR*-ITD can be suggested by DNA-methylation profiling when the methylation class (MC) is HGNET-BCOR (14). This technique which uses arrays to determine DNA methylation patterns across the genome is a powerful method for CNS tumor classification (26). It is possible to upload the raw IDAT files to https://www.molecularneuropathology.org for supervised analysis using the Random Forest methylation class prediction algorithm and copy number profiles (26). Recently, DNA-methylation profiling was used for classifying extracranial sarcomas, including those with *BCOR* alteration. Accordingly, raw IDAT files can be uploaded on https://www.molecularsarcomapathology.org). However, careful attention must be paid to the common calibrated score threshold. Thresholds may be set at 0.84 or 0.90 (27). A confident diagnosis can usually be made with scores above the threshold value, whereas caution is advised with a lower score. In this case, the pathologist should take into account the diagnosis suggested by the classifier, the clinicopathological information, and if indicated, additional techniques should be performed to reach the right diagnosis. DNA-methylation profiling was performed in all cases with a presumptive pathological diagnosis of HGNET-BCOR. Except for the three cases previously published using 450K probes and an older version of the classifier (16), all cases with *BCOR*-ITD detected by our dPCR assay displayed a calibrated score of 0.99 for the MC HGNET-BCOR. In two cases that were not duplicated by dPCR the score for HGNET-BCOR methylation class was 0.57 and 0.85; these cases demonstrated by RNA sequencing *EP300-BCOR* fusion and *KDM2B-NUTM2* fusion, respectively. *EP300-BCOR* fusion has been recently reported in a group of children with low-grade gliomas displaying divergent histological features including pilocytic astrocytoma or dysembryoplastic neuroepithelial tumor (28). These features were absent in our case which displayed perivascular pseudorosettes, monotonous round to oval nuclei with fine chromatin, numerous mitotic figures and necrosis, and neither GFAP nor synaptophysin expression in accordance with our presumptive diagnosis of HGNET-BCOR. This case, which has been previously published (21), demonstrates strong similarities regarding clinico-radiological, histopathological, immunohistochemical, and methylome features with HGNET-BCOR with ITD but its classification according to the c-IMPACT-NOW update 6 (15) will be difficult since the terminology of “CNS tumor with *BCOR*-ITD” cannot be used in the absence of *BCOR*-ITD. The other case that harbored *KDM2B-NUTM2* fusion was of particular interest since the score of HGNET-BCOR methylation class was very high. Importantly, *KDM2B* is part of the non-canonical variants of *PRC1*: the *PRC1.1*. PRC1.1’s core components include RING1A/B and PCGF1 which form a heterodimer for PRC1 assembly. BCOR associates with the complex by binding to PCGF1 through its PUFD domain. PCGF1 also interacts with KDM2B which recognizes non-methylated CpG islands and is responsible for the recruitment to the chromatin of the PRC1.1 complex (3). Therefore, we can postulate that in CNS tumors, *KDM2B-NUTM2* fusion might generate a DNA-methylation signature mimicking the one generated by *BCOR*-ITD alteration. Of interest, this tumor shares with CNS tumor with *BCOR*-ITD similar pathological features. CNS tumor with *BCOR*-ITD is a new tumor type belonging to the category of embryonal tumors and is not considered as a mesenchymal tumor in contrast to other tumors with *BCOR*-ITD outside the CNS, although it shares with these tumors the same minimally duplicated region in the *BCOR* gene. In the present series of 13 *BCOR*-ITD cases, we observed that the fractional abundance was higher in CNS tumor with *BCOR*-ITD than in other tumor types except for one case reported as URCS but occurring in the cerebello-pontine angle. Whether this case occurring in a 26 year-old patient is a true sarcoma or a CNS tumor with *BCOR*-ITD remains unsolved. Altogether, the examples reported highlight that even with typical clinico-pathological features (including BCOR immunohistochemistry) or a DNA-methylation score suggestive of HGNET-BCOR or sarcoma with *BCOR* alteration, the confirmation of *BCOR*-ITD by an appropriate technique is mandatory. Specifically, we recommend dPCR for the detection of the *BCOR*-ITD because this technique is rapid, cost-effective and sensitive with a short turnaround time. In the case of a negative result (lack of *BCOR*-ITD), RNA-sequencing is the more appropriate technique. In addition, RNA-sequencing is indicated when the clinicopathological features or DNA-methylation score are not typical. The recommended diagnostic workflow is summarized in **Figure 5**.

## Conclusion

In the era of histomolecular classification of human tumors, the need to develop appropriate molecular tests is becoming increasingly important. The dPCR assay that we have set up is of particular interest to detect *BCOR*-ITD since it is a cheap, robust, 100% sensitive, and specific test that can be largely used in various cancers known to share this genetic alteration.

## Data Availability Statement

The raw data supporting the conclusions of this article will be made available by the authors, without undue reservation.

## Ethics Statement

The studies involving human participants were reviewed and approved by the APHM ethics committee. Written informed consent to participate in this study was provided by the participants’ legal guardian/next of kin.

## Author Contributions

FF, RA, DF-B, and CC supervised the study and wrote the manuscript. MH, MB, CB, GC, FL, NM, RP, DP, AR, AT-E, PV, and AV contributed to build the tissue cohorts by providing precious tumor samples (FFPE material) and related available data. DF-B and RA made the selection of the samples and performed pathological review. FF, DB, and RA designed the *BCOR*-ITD digital PCR detection assay, they performed the digital PCR assay and contributed to data analysis. All authors contributed to the article and approved the submitted version.

## Funding

We thank the ARTC-Sud patients’ association (Association pour le Recherche sur les Tumeurs Cérébrales), the Association Cassandra, the Association Liv&Lumière, the Imagine For Margo Association, Enfants Cancers Santé (ECS), the SFCE (*Société Française de Lutte contre les Cancers et Leucémies de l’Enfant et de l’Adolescent*) the Cancéropôle PACA, and the GIRCI Méditerranée (GlioMark protocol) for their financial support. We thank the RENOCLIP-LOC network (Réseau national de neuro-oncologie clinico-pathologique pour les cancers rares du système nerveux central) funded by the French Institut National du Cancer (INCa) grant (Decision n°2019-29).

## Conflict of Interest

FF is Scientific Director of ID-Solutions, Grabels, France.

The remaining authors declare that the research was conducted in the absence of any commercial or financial relationships that could be construed as a potential conflict of interest.
